# From Controller to Screen: A Narrative Review of Video Games in Medicine

**DOI:** 10.7759/cureus.82745

**Published:** 2025-04-21

**Authors:** Mohammed S Rais, Eric M Schorr, Matthew B Winfield, Farhana S Rais, Mohammed S Rais

**Affiliations:** 1 School of Medicine, Louisiana State University Health Sciences Center, New Orleans, USA; 2 Cardiology, Cardiovascular Institute of the South, Thibodaux, USA

**Keywords:** cardiovascular health, health, health wellness, job stress, medical burnout, mental cognition, mental health, surgery, surgical dexterity, video games

## Abstract

Video games have become a global phenomenon in modern society as a means of entertainment, social interaction, and stress relief. Although they are often associated with negative effects, such as a lack of productivity and a sedentary lifestyle, research on video games displays potential benefits in everyday life and medical training. This narrative review provides an overview of video games and their contributions to stress relief, surgical dexterity, and cardiovascular health in the context of medical education and general wellness. To conduct this study, a comprehensive literature review search was performed utilizing PubMed and Google Scholar, placing emphasis on studies that discussed video games in society, in medicine, and in association with health. The following search terms were included: “video games in medicine,” “video games and mental health,” “video games and cardiovascular health,” “video games and surgery,” and more. Exclusion criteria for this review included papers not written in the native English language and case reports due to inherently small sample sizes.

Video games show promise in relieving stress and increasing cognition, improving surgical dexterity and hand-eye coordination, and improving general cardiovascular health. They pose as a potential implementation to improve the health and wellness of medical trainees. Despite the negative societal implications of video games, there is evidence that they provide health benefits and help hone skills needed in medicine. By reducing stress, preventing long-term disease, and increasing surgical skills and dexterity, video games tap into factors from which all people, especially physicians and medical trainees, can benefit from.

## Introduction and background

Video games have spread worldwide and can be played by any cognitively inclined age group. With the advancements of technology as well as increased affordability, video games can be found in numerous households throughout the world. An estimated 90% of children over the age of two years play video games, and roughly three-quarters of households in America own a video game console [1]. Numerous devices like consoles, such as the PlayStation (Tokyo, Japan: Sony Interactive Entertainment) and Xbox (Redmond, WA: Microsoft Gaming), handheld devices, such as the PlayStation Vita and Nintendo Switch (Kyoto, Japan: Nintendo), as well as non-mobile personal computer (PC) gaming are commonly found in many households throughout the world [1,2]. Video games have garnered a negative reputation in society due to their addictive potential and ability to distract individuals from school, work, and other pertinent tasks [2,3]. However, research shows that video games provide substantial benefits in a variety of domains, not only in everyday life but also in the fields of medicine and general health and wellness [4,5].

Medical training is often understood as a crucial time, in which medical school graduates become clinicians and work to hone their knowledge and skills as physicians before becoming attending physicians. Medical school and residency programs are known to be intensive due to the stringent requirements of learning, studying, and ultimately practicing to best take care of patients and best help the medical team. There are numerous studies that discuss burnout in medicine; existing literature consisting of systematic reviews and randomized controlled studies points out a correlation between video game usage and reduced stress as well as improved mental health [5-8].

In surgery, dexterity and hand-eye coordination are crucial for incising, maneuvering, suturing, and knot-tying. Playing video games is correlated with improved dexterity in surgeons in simulated trials [9]. This narrative review delves into the positive effects of video games in medicine from the perspectives of medical training and overall health.

## Review

Methods

To conduct this study, a comprehensive literature review search was performed utilizing PubMed and Google Scholar, placing emphasis on studies that discussed video games in society, in medicine, and in association with health and wellness. The following search terms were included: “video games in medicine,” AND “video games and mental health,” AND “video games and cardiovascular health,” AND “video games and surgery,” AND “video games in medical training.” Exclusion criteria for this review included papers not written in the native English language and case reports due to inherently small sample sizes that do not yield enough clinically relevant data.

Discussion

Video games have become a global phenomenon due to the growing demand for technological entertainment and increased accessibility worldwide. With the ability to engage in an interactive form of media, with new friends online or those known in real life, the fantastical and collaborative nature of video games makes gaming, especially while bonding with friends, enticing [10]. Like social media, video games are another avenue for humans to socialize, as humans are biologically rooted in being social creatures, desiring companionship with others [11]. The “fun” behind video games lies in the emotional experience attached to playing the game initiated by the reward system in the brain. The dopamine released in the brain while playing video games increases feelings of pleasure, satisfaction, and motivation that keep players engaged in continuing to play the game [12]. With video games becoming more commonplace, the intersection of video games with mental health, physical health, and overall utility in medicine deserves to be explored further.

Video games’ impact on mental health and cognition

Video games have a complex and multifaceted impact on mental health and cognition, with research highlighting both potential benefits and risks. One notable advantage is the ability of video games to enhance cognitive function, particularly in areas such as attention, memory, and problem-solving. Research has shown that children who play video games regularly exhibit improvements in cognitive performance, particularly in tasks requiring attention and response inhibition, compared to their non-gaming peers [13]. Additionally, action video games have been shown to improve visuospatial skills, as they require players to track multiple objects, navigate complex environments, and make quick decisions under pressure. These gamers also had improved visual attention over both the central and peripheral visual fields compared to non-gamers [14]. This cognitive enhancement has significant implications for medical professionals, particularly surgeons and emergency medicine physicians, whose work requires rapid decision-making and precise hand-eye coordination. Some studies suggest that video game training may even improve surgical performance, making it a potential tool for medical education [15].

Beyond cognition, video games also play a significant role in mental health, with some studies suggesting that casual gaming can be an effective tool for stress relief and mood regulation. Research has found that casual video games, such as puzzle and simulation games, significantly reduced stress and improved mood among players [16]. These effects are particularly relevant for medical professionals, who often face high levels of stress and burnout. While gaming can offer cognitive engagement and relaxation, its impact on mental health varies depending on individual differences and gaming habits. Excessive gaming has been linked to negative mental health outcomes, particularly when it interferes with daily life. Studies have associated prolonged gaming with increased risks of anxiety, depression, and social withdrawal, particularly among individuals predisposed to these conditions [17]. Research has found that while video games can improve cognitive function, structural differences in the brains of frequent gamers suggest alterations in reward processing, which may contribute to compulsive gaming behaviors. Specifically, gamers in a study showed heightened activity in the dorsolateral prefrontal cortex (DLPFC) during cognitive control tasks. The DLPFC plays a crucial role in working memory, strategic planning, and problem-solving, which suggests that gaming may enhance these executive functions. This aligns with previous research showing that video games, particularly action and strategy games, can improve multitasking skills, cognitive flexibility, and task-switching abilities. However, this study also raised some concerns about the potential downsides of these changes. While some gamers displayed improved cognitive control, others exhibited reduced activation in the anterior cingulate cortex (ACC), which is a region involved in monitoring and regulating impulsive behaviors. Lower ACC activity has been associated with risk-taking behaviors and compulsive tendencies, which may explain why some individuals develop problematic gaming habits or gaming disorders [18]. This is particularly relevant for medical professionals, as problematic gaming may contribute to sleep disturbances, fatigue, and reduced professional performance.

Ultimately, video games are neither inherently good nor bad for mental health and cognition. Their effects depend on various factors such as game type, duration of play, and individual susceptibility. While research supports the cognitive and emotional benefits of gaming, excessive use may contribute to mental health challenges, underscoring the importance of balance. As video games continue to evolve, further research is needed to optimize their use for cognitive enhancement and therapeutic purposes while mitigating their potential risks. Medical professionals, in particular, should recognize both the benefits and drawbacks of gaming, whether as a cognitive training tool, a stress-relief method, or a potential source of distraction. Understanding these dynamics will allow healthcare providers to better counsel patients, as well as manage their own well-being in an increasingly digital world.

Video games’ impact on cardiovascular health and overall wellness

The correlation between video games, cardiovascular health, and overall wellness is noteworthy. Research commonly shows that physical activity, smart dietary decisions, and healthy lifestyle choices are good for a person’s overall longevity [19]. A sedentary lifestyle is associated with increased adverse health conditions such as cardiovascular disease, hypertension, diabetes mellitus, and more [20]. Because most modalities of video games often require one to sit in front of a television screen or computer for extended periods of time, video games are commonly associated with more negative health risks [21]. However, recent literature reflects contrary findings in that certain types of video games actually promote cardiovascular health benefits.

The act of playing video games is often seen as “stress relieving,” making it a common activity for students, workers, and anyone who suffers from above-average stress levels. Playing video games can lead to a decrease in cortisol levels, which is a physiologic hormone produced in the adrenal gland that helps regulate blood pressure, blood glucose, stress response, immune response, and sleep [22,23]. In times of stress, the levels of cortisol are elevated in the bloodstream, and this elevation, in the long term, is associated with an increased cardiovascular disease profile with strong associations with coronary artery disease and acute coronary syndrome [24]. With heart disease being the number one cause of death in America, implementing video games can be an easy step in preventing future cardiovascular deficits [25].

Video games come in different shapes, sizes, and modalities. For instance, exergames are video games that require physical activity in order to be played [26]. They are correlated with increased cardiovascular health due to not only physical activity but also player retention in terms of increased playtime due to the fun and immersive nature of these games, allowing players to focus on the activities of the game and without actively realizing the benefits of exercise simultaneously [26,27]. Many active video games, such as the Wii, Just Dance, and numerous VR simulation games, have shown an adequate effect in increasing heart rate and metabolic activity, as playing these games mimics exercise [28]. These findings are especially crucial to medical professionals and medical trainees, as their time is limited, and burnout rates are high [29]. For doctors in training, video games offer a great deal of benefits, such as improving cognitive function and memory, increasing surgical skills and dexterity, and serving as a method of relaxation. Individuals can also see long-term health benefits, especially with video games that require physical movement. Although these studies do not include study groups consisting of medical professionals, the addition of video games in the medical field can help tap into benefits for both productivity and overall health and well-being in the medical field.

In the pediatric population, exposure to exergames has been a tool to improve early activity and to build healthy habits [30]. Exergames allow children to hone in on a safe, active, and enjoyable means of entertainment while additionally providing health benefits from engaging in physical activity. Studies show that exergames in the pediatric population, due to their ability to mitigate sedentarism, have promising effects in preventing childhood obesity [30]. This finding is crucially important as childhood obesity affects around 17% of children in America [31]. Children diagnosed with childhood obesity are more likely to remain obese into adulthood. Obesity in children predisposes them to comorbidities such as diabetes mellitus and various cardiovascular ailments at younger ages and into adulthood. Obesity in childhood is associated with increased morbidity and premature deaths. Increasing activity in this population would help mitigate long-term negative effects in the pediatric population [32]. Exergames pose as a safe, fun, and viable solution in getting the pediatric population more active to ward off future disease risk.

Video games are linked to improved surgical skills and dexterity

Manual dexterity refers to the ease, skill, and grace with which an individual uses their hands [33]. Manual dexterity results from a myriad of complex physiologic relationships between the peripheral nervous system, intrinsic and extrinsic muscles of the hand, and the small bones of the hand coalescing into fine motor function [34]. Among other skills, manual dexterity is invaluable within the operating room. Activities such as suturing, knot-tying, and, more recently, surgical simulations are all commonly used to maintain and refine surgical dexterity [35].

With the invention of video games in 1967, many people today likely grew up with gaming experience [36]. Though these games were likely played as a pastime or out of competition, they may have served a beneficial role in a handful of skills in the future surgeons’ careers. A 2017 study by Alsafi et al. explored the relationship between computer-based gaming experience and manual dexterity. In the study, 50 students aged 18-22 years completed a questionnaire on their exposure to computer games. The students were shown a video demonstrating renal artery cannulation and were then asked to reproduce the procedure. The study found that students who played computer games >10 hours per week had better dexterity scores than those who did not play computer games - 9.1 versus 10.2 seconds (p=0.0237) [9]. However, dexterity is not the only surgical skill in which video games have provided a positive influence. The use of computer games correlates with better precision of arm-hand movements, lower time of errors, and a shorter total time of performing tasks [37].

With advancing surgical technologies in minimally invasive procedures, computer games continue to provide a beneficial impact on skills needed in performing surgery. A study by Gupta et al. showed that a history of gaming and video-game-based training correlated with improved metrics in laparoscopic tasks such as maintaining camera horizon, complex ball manipulation accuracy rates, and shorter completion time of a complex laparoscopic simulator task [4]. As technology in surgery continues to advance, surgical training must continuously adapt. Future research should be dedicated to implementing beneficial aspects of computer-based gaming into surgical training in order to maintain the necessary skills in an ever-changing environment.

Gamification of medical education

The idea of “gamifying” medical education has garnered positive attention, as it increases student attention, engagement, and performance. The reason for this lies in the similar effects of video games by incorporating the chance to win rewards and compete among peers. This “gamification” acts similarly to video games as it taps into the brain’s reward system pathway, increasing the release of dopamine and making studying, which is often daunting for most students, more fun and enjoyable [38]. 

One anecdotal example is the use of “Kahoot!” in the classroom. “Kahoot!” is a game-based learning platform in which students answer questions as fast as they can correctly to score the most points and win the game overall [39]. This game-based learning platform increases excitement with learning, as students focus their attention on the game aspect of “Kahoot!” while making the actual study and recall feel less like a chore. In addition, results in a review of studies have shown the use of "Kahoot!" to show increased class attendance, fewer late arrivals to class, higher downloads of pertinent course materials, improved classroom dynamics, and higher final grades when compared to non-Kahoot! cohorts [40]. In addition, this game also promotes collaboration, as teams can be formed with multiple students, and they can work together as a team to win the game.

## Conclusions

Overall, the implication of video games, both in daily life and in the medical field, shows great promise when used appropriately. Video games that require the user to move to play offer enjoyment, mental alleviation, and exercise, which all synergistically help to reduce stress and burnout from the demands of clinical duties. Future longitudinal, randomized control trials and simulation studies are needed to obtain more data to garner enough favor for video games to play a larger and more formative role in the process of medical training. The positive impacts made at this level has the ability to pay dividends for the future healthcare system of the United States by mitigating long-term disease and decreasing disease burden.
